# An alphavirus-based therapeutic cancer vaccine: from design to clinical trial

**DOI:** 10.1007/s00262-018-2276-z

**Published:** 2018-11-21

**Authors:** Amrita Singh, Georgia Koutsoumpli, Stephanie van de Wall, Toos Daemen

**Affiliations:** 1 Department of Medical Microbiology, Tumor Virology and Cancer Immunotherapy, University of Groningen, University Medical Center Groningen, HPC EB88, PO Box 30.001, 9700RB Groningen, The Netherlands; 2 Present Address: Radiotherapy and OncoImmunology Laboratory, Department of Radiation Oncology, Radboud Institute for Molecular Life Sciences, Radboud University Medical Center, Nijmegen, The Netherlands

**Keywords:** Therapeutic vaccine, Recombinant Semliki Forest virus vector, Hepatitis C virus, Human papillomavirus, Cervical intraepithelial neoplasia, PIVAC 17

## Abstract

Cancer immunotherapy has greatly advanced in recent years. Most immunotherapeutic strategies are based on the use of immune checkpoint blockade to unleash antitumor immune responses or on the induction or adoptive transfer of immune effector cells. We aim to develop therapeutic vaccines based on recombinant Semliki Forest virus vectors to induce tumor-specific effector immune cells. In this review, we describe our ongoing work on SFV-based vaccines targeted against human papillomavirus- and hepatitis C virus-related infections and malignancies, focusing on design, delivery, combination strategies, preclinical efficacy and product development for a first-in-man clinical trial with an HPV-specific vaccine.

## Introduction

Immunotherapy, which was recognized as the breakthrough of the year 2013, has given a new direction to the development of anti-cancer therapies. Cancer immunotherapies aim to stimulate or unleash the patient’s immune system to attack cancers. These strategies are primarily based on antibodies targeting immune checkpoints, tumor-specific antibodies, a diversity of adoptive cell therapies, therapeutic vaccines and immunomodulators [1, 2]. Therapeutic vaccines include synthetic peptides, recombinant proteins, nucleic acids, autologous cells and bacterial or recombinant viral vectors. Recombinant viral vector vaccination is attractive above all others as it generally offers high infection efficiency, antigen expression and immunogenicity. Vector-based vaccines have been generated from a diversity of viruses including adenoviruses, adeno-associated virus, vaccinia viruses, poxviruses and alphaviruses [2]. Our work in recent years has focused on developing therapeutic cancer vaccines based on Semliki Forest virus (SFV) replicon particles for the treatment of human papillomavirus (HPV)- and hepatitis C virus (HCV)-related infections/malignancies.

This review aims to summarize the development of our SFV-based cancer vaccines. We will describe the various preclinical evaluation studies conducted in our lab, which ultimately led to the first-in-man trial of an SFV-based vaccine in 2017.

## The Semliki Forest virus replicon vector system

The most frequently used alphavirus vectors are derived from SFV [3], Venezuelan Equine Encephalitis virus (VEE) [4] and Sindbis virus (SIN) [5]. Alphaviruses are enveloped viruses containing a positive strand RNA genome of about 11–12 kb, coding for the non-structural replicase and the structural proteins. The genomic RNA has a methylated nucleotide cap at the 5′-terminus and a polyadenylated tail at the 3′-terminus, resembling cellular mRNA. Alphaviruses form spherical enveloped particles (65–70 nm diameter) with their genome contained within an icosahedral capsid. The envelope contains two major glycoproteins, E1 and E2, forming heterologous spikes that act as attachment proteins. After virus attachment and entry into the cell, gene expression and replication take place within the cytoplasm [6, 7].

**Fig. 1:**
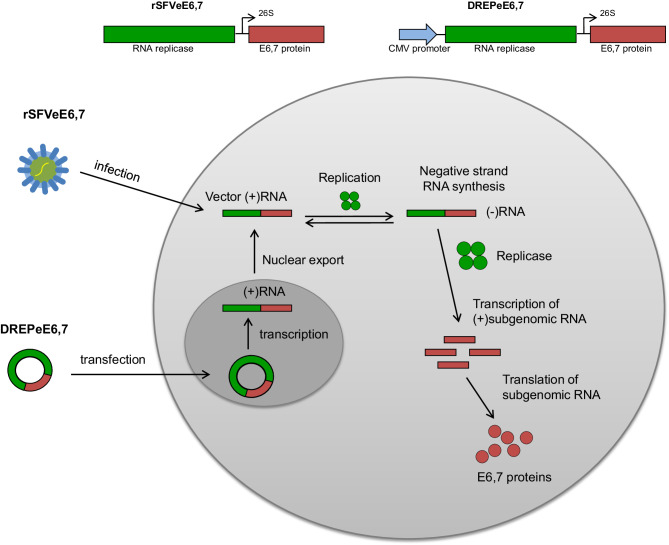
Production of antigen of interest by SFV replicon. Infection with rSFVeE6,7 results in introduction of vector RNA directly in the cytoplasm. Thereafter, the RNA translates the SFV replicase which drives the transcription/amplification of replicon RNA. Translation of large amounts of subgenomic RNA results in production of the encoded fusion protein. Upon delivery of DREPeE6,7 into the cell, DNA is transported to the nucleus where transcription of the replicon takes place following which produced RNA is exported to the cytoplasm followed by the same replication and translation process as described above for rSFVeE6,7

The SFV-based replicon vector system was first developed by Peter Liljeström and Henrik Garoff at the Karolinska Institute, Stockholm [3]. Additional SFV-based vectors were developed with improved biosafety [8, 9]. The recombinant SFV (rSFV) vector RNA codes for the viral replicase while the sequence encoding the structural proteins of SFV can be replaced by a gene of interest. The recombinant virus particles called replicons, due to their self-replicating nature, are obtained by co-transfection of cells with the vector RNA and the helper RNA encoding the structural proteins [10]. As the helper RNA does not express the replicase, amplification of both the vector and the helper RNAs is driven by the replicase of the vector RNA. The capsid protein recognizes the packaging signal located in the replicase region of the vector RNA and packs only the recombinant vector RNA into nucleocapsids. The envelope/spike proteins migrate to the plasma membrane where they interact with nucleocapsids leading to the formation and subsequent budding of virus replicon particles (Fig. 1). Since SFV vectors are engineered such that the replicon particles lack sequences coding for the viral structural proteins, they are capable of only one round of infection and replication. SFV RNA does not integrate into the host genome and since infection with SFV induces apoptosis, this minimizes the risk of constitutive expression of heterologous proteins (antigen of interest). For clinical translation, increased biosafety is required to further reduce the risk of recombination of the two RNAs that could lead to the formation of replication-competent virus. For this, Smerdou and Liljeström developed the so-called two-helper system in which the structural genes coding for the spike and capsid proteins are placed on two separate helper RNA constructs [9].

Following immunization, rSFV particles infect a broad range of cells that undergo apoptosis. Thereafter, apoptotic bodies containing antigen are taken up by antigen-presenting cells (APCs) which can then present to CD8^+^ and CD4^+^ T cells leading to the generation of antigen-specific cellular immune responses. A major difference between VEE and SFV replicon particles is that VEE replicon particles preferentially target dendritic cells resulting in direct MHC class I presentation of the antigen, whereas SFV replicon particles cannot translate their RNA in APCs but induce CTL response via cross-priming [11, 12]. Infection with SFV and other alphaviruses leads to the formation of double-stranded RNA (dsRNA) intermediates, which are recognized as “danger signals” by pattern recognition receptors such as RIG-I and trigger production of type I interferons [13, 14]. Therefore, recombinant vector vaccines based on alphaviruses are capable of activating both the innate and the adaptive arms of the immune system.

Another type of SFV-based vector that Liljestrӧm and colleagues developed is the DNA replicon (DREP) [15]. This vector system combines the advantages of the SFV replicon vector with that of a DNA vaccine, which are ease of production and cost-effectiveness. The plasmid contains cDNA sequences of the SFV replicase, where the transcription of the viral replicon is under the control of a CMV promoter and where the mRNA coding for the foreign antigens is expressed from an internal viral promoter on the replicon RNA. Upon transfection with DREP plasmid DNA, the DNA is transported to the nucleus where the RNA polymerase drives its transcription to RNA. The recombinant mRNA transcript is then transported to the cytoplasm, where the translation of the SFV replicase directs replication and high production of the foreign protein in a way similar to that by SFV replicons [10].

## Preclinical evaluation of SFV-based replicon vaccines

### Therapeutic HPV vaccine rSFVeE6,7

HPV infection is associated with over 99% cases of cervical cancer, the fourth most common cancer type among women worldwide [16, 17]. HPV infection is also a causal agent of other genital cancers such as penile, vaginal, vulvar and anal but also an increasing percentage of oropharyngeal cancers. The high-risk HPV types 16 and 18 are the most common and are associated with approximately 70% of cervical cancer cases. In general, the virus is cleared but if the infection persists, low- to high-grade cervical intraepithelial neoplasia (CIN) and ultimately cervical carcinoma may develop. Malignant transformation of epithelial cells is accomplished through integration of HPV viral DNA genome into the host genome with the disruption of genes including E2, a negative regulator of the HPV oncoproteins E6 and E7. High expression of these oncoproteins in transformed cells is responsible for the induction and maintenance of cellular transformation.

Currently, there are three prophylactic HPV vaccines available: Cervarix, Gardasil and Gardasil-9 [18]. These vaccines effectively induce HPV type-specific antibodies to prevent the most prevalent high-risk HPV infections. Despite the availability of these highly effective prophylactic HPV vaccines, over 85% of young adolescent females worldwide are not vaccinated [19], either because an HPV vaccine is not provided in a national vaccination program in many low- or middle-income countries or because of fear of side-effects or opposition to vaccination.

In patients with (pre)malignant cervical cancer, cellular and humoral responses are observed, but they are not sufficient to eliminate HPV-transformed cells [20]. A therapeutic vaccine that is able to induce strong cellular immune response against HPV-transformed cells is therefore highly desirable. Since the viral oncoproteins E6 and E7 of HPV are constitutively expressed in transformed cells, they serve as ideal targets for immunotherapy against HPV-induced lesions or malignancies. We first generated an SFV vector expressing the E6 and E7 protein of HPV16 as separate proteins, rSFVE6E7. The replicon particle vaccine based on this vector induced moderate immune responses in a murine model of cervical cancer with TC-1 tumors [21]. To enhance this response, we inserted the first 34 amino acid residues of the capsid gene of SFV, which functions as a translational enhancer [22], in front of the gene coding for a fusion protein of E6 and E7. Thus, a recombinant SFV vector expressing high levels of a stable fusion protein of HPV16 E6 and E7 was generated, termed rSFVeE6,7 [23]. We demonstrated that rSFVeE6,7 replicon particles could induce more potent anti-tumor responses than the initial vaccine rSFVE6E7, leading to the complete eradication of established TC-1 tumors. Furthermore, tumor rechallenge studies demonstrated long-term protection. When previously immunized mice were rechallenged with tumor cells after 3 or 6 months, the mice were still protected from tumor outgrowth without another round of immunization and high levels of CTL activity could be measured even up to 11 months after immunization [23, 24]. rSFVeE6,7 vaccination could also break immune tolerance in HPV16-transgenic mice [25].

Various immunotherapeutic approaches use viral vector-based systems for immunization against tumors. The adenovirus (Ad) system is one of the most widely used viral vector system, which has also been extensively studied in human trials [26–29]. We therefore compared the efficacy of a recombinant replication-defective Ad (rAd) vaccine expressing the fusion protein of E6 and E7 with the rSFVeE6,7 vaccine. We found that prime-boosting with rSFVeE6,7 resulted in higher CTL activity and anti-tumor activity compared to that with rAdeE6,7, even with 100- to 1000-fold lower doses compared to the adenoviral vector [30]. Part of this result can be ascribed to the fact that the low dose of rSFVeE6,7 required to induce an optimal immune response (< 10^6^ infectious particles), generated relatively low levels of SFV-neutralizing antibodies that did not prohibit the responsiveness to a booster immunization. The levels of neutralizing antibodies elicited upon rAdeE6,7 immunization however completely blocked antigen expression and a booster response. Vector-specific immunity against adenoviral vectors are extensively studied and strategies are being developed to reduce anti-Ad immunity to improve their efficacy as gene delivery system [31, 32].

### Therapeutic hepatitis C virus vaccines

Hepatitis C virus (HCV) is a human tumor-associated virus that is transmitted by blood and mainly infects the liver. The majority of hepatitis C virus-infected patients (> 70%) do not clear the infection naturally and develop chronic hepatitis, which is associated with the development of liver cirrhosis, liver failure and hepatocellular carcinoma [33]. The standard-of-care treatment for HCV infection in developed countries is based on a combination of very effective antiviral drugs. Although these drugs are highly potent, they are very expensive, may induce resistance and are not widely available. It is also important to note that antiviral treatments aim only at inhibiting HCV replication, but not at inducing HCV-specific T-cell responses and protective immunity, which has been reported to be crucial for the eradication of HCV-infected cells.

In patients with chronic HCV infection, a poor and narrow-spectrum cellular immune response against HCV is often observed while in patients who recovered from acute infection, the immune responses are broader. Thus, boosting the host’s immune response against multiple antigenic epitopes would theoretically be a valid strategy to eradicate persisting viruses and halt associated liver diseases. In the context of hepatitis C virus (HCV) infection, the non-structural proteins (nsPs) of HCV, which are considered to be immunogenic and genetically conserved, are identified as promising targets for vaccine development. With a view to broaden the spectrum of T-cell responses [34], a transgene coding the entire non-structural region of HCV could allow for responses against subdominant epitopes as well, contributing to a wider T-cell repertoire [35].

We therefore explored the possibility of expressing the HCV nsPs in the rSFV vector to promote the generation of HCV-specific and broad-spectrum T-cell responses. Incorporation of the major part of the nsPs of HCV, as large as 6.1 kb, did not affect the immunogenicity of the vaccine. We showed that rSFV vectors expressing HCV NS3/4A (rSFVeNS3/4A) or all nsPs of HCV (rSFVeNS2′-5B′) were capable of inducing strong NS3-specific CD8^+^ T-cell responses, although the response was slightly lower with rSFVeNS2′-5B′ [36]. Both vectors induced polyfunctional CD8^+^ T cells, exhibited HCV-specific cytolytic activity both in vitro as well as in vivo and induced delay in tumor growth.

Taken together, preclinical evaluation of replication-deficient rSFV vectors demonstrated the ability to induce strong effector and memory CTL responses in immunocompetent as well as immune-tolerant mice and to eradicate pre-established HPV-transformed tumors and HCV-infected cells.

## Anti-vector responses

As previously mentioned, immunization with replicon viral particles may induce vector-specific immunity, which in turn could influence the efficacy of subsequent booster immunizations. We investigated the effect of vector-specific immune responses on transgene expression and CTL activation by rSFV. We demonstrated that passively transferred SFV-neutralizing antibodies did not prohibit transgene-specific CTL responses, despite the fact that the transgene expression was reduced, albeit not to the level observed with adenoviral booster injections [30, 37]. On the other hand, priming with irrelevant rSFV reduced transgene expression upon subsequent administration of rSFV and inhibited CTL induction due to vector-specific responses, which could be reversed with the co-administration of a relevant antigen (E7-virosomes) at priming. Incorporating an E7-protein antigen along with irrelevant rSFV and then boosting with rSFVeE6,7, induced a similar percentage of E7-specific CTLs as prime-boost immunization with rSFVeE6,7. This points towards a possible role of T-cell competition as a mechanism by which vector-specific immunity interferes with transgene-specific CTL induction. This study confirmed that virus-neutralizing antibodies or vector-specific CTLs do not prohibit the boosting efficacy of rSFV in homologous prime-boost immunizations and rather T-cell competition has a stimulatory effect in such regimens [37]. Yet, SFV-based vaccines have also been successfully used in heterologous combinations with other vaccines [38–42].

## Antigen design

As the immunogenicity of rSFV relies on cross-priming, the protein of interest should not be (completely) degraded before being processed by antigen-presenting cells. As described above, expression of a more stable fusion protein of E6 and E7 induced higher immune responses compared to the vaccine expressing the E6 and E7 proteins separately [21, 23, 24]. With the aim of further improving the efficacy of our vaccine, we assessed the potential of the replicon particles expressing the HPV16 E6,7 fusion protein or E7SH (a shuffled version of the E7 protein) coupled with helper T-cell epitopes and an ER targeting signal (sigHELP-KDEL), a strategy which successfully improved the efficacy of DNA vaccines [43, 44]. Immunization with both rSFVe-sigHELP-E6,7-KDEL and rSFVe-sigHELP-E7SH-KDEL augmented E7-specific T-cell responses as compared to the corresponding parent vectors. A suboptimal dose of rSFVe-sigHELP-E6,7-KDEL significantly enhanced the anti-tumor responses compared to rSFVeE6,7. Strong memory responses were generated providing protective immunity to the mice against formation of TC-1 tumors.

Inclusion of helper T epitopes and an ER targeting signal further increased the immunogenicity of our HPV vaccines, which was likely due to enhanced protein stability and rendered therapeutic as well as prophylactic immunity with a suboptimal dose of the vaccine [45].

## Delivery routes and methods

The route of immunization influences vaccine efficacy. We demonstrated that subcutaneous (s.c.) injection of the vector vaccine elicits potent CTL and anti-tumor responses [24]. We further explored different routes of immunization to determine their influence on the degree of vaccine-induced immune response. We demonstrated that these responses were strongly augmented with i.v. (intravenous) and i.m. (intra-muscular) administrations. Although CTL activity was induced upon i.p. (intraperitoneal) and s.c. immunizations, i.v. and i.m. induced much higher CTL frequencies. We also compared i.m. vs i.v. immunization with two suboptimal treatment regimens and concluded that both i.m. and i.v. administrations are equally effective in generating anti-tumor responses [46].

Besides the above-mentioned delivery routes, we examined intra-dermal delivery by means of tattooing. The skin, being rich in APCs, such as Langerhans cells and dermal dendritic cells, is an attractive site for immunization. Tattooing has been mostly described for DNA- and peptide-based vaccines [47–50]. We compared tattoo delivery vs intra-muscular administration of rSFVeE6,7 and found that despite having a tenfold lower overall transgene expression (injection site and draining lymph nodes), tattooing induced similar levels of immune responses in comparison with i.m. delivery of the replicon particles [51]. This delivery method resulted in potent therapeutic anti-tumor response and stimulated long-lasting memory T cells, establishing high immunostimulatory potential of intra-dermal delivery with regard to recombinant SFV vector vaccines.

Following on this route, we generated an HPV16 E6,7 vaccine based on the DREP vector as described above [15] and compared it with a conventional DNA vaccine [52]. The DNA vaccines were administered intra-dermally followed by in vivo electroporation [53, 54] resulting in strong anti-tumor effects with the DREP vaccine when compared with the conventional DNA vaccine pVAX. Upon administration of equimolar doses of DREP and pVAX, we found that DREP strongly enhanced the expression of the protein and high percentages of E7-specific CD8^+^ T cells were induced, which rendered effective anti-tumor immunity compared to pVAX. Remarkably, even with a dose 1000-fold lower than that of the conventional DNA vaccine, potent anti-tumor efficacy was observed [52]. On the whole, intra-dermal delivery of DREP along with electroporation as a method of augmenting its efficacy could be a promising alternative of vaccine administration for favorable clinical outcome.

## Combination strategies

Tumors adopt various mechanisms to evade the immune system such as immune-suppressive cells, upregulation of inhibitory signals such as PD-1 [55], CTLA-4 [56], etc. Therefore, it would be beneficial to target these mechanisms to avert their influence on anti-tumor immunity and improve the suppressive tumor microenvironment. With the purpose of enhancing the efficacy of therapeutic immunization with rSFVeE6,7, the next step was to combine the vaccine with strategies that can modulate the tumor environment.

We investigated two immune-suppressive cell populations, regulatory T cells (Tregs) and myeloid-derived suppressor cells (MDSCs). In patients with cervical intraepithelial neoplasia (CIN) and cervical cancer, increased frequencies and immunosuppressive activity of Tregs were observed [57]. Furthermore, therapeutic immunization with different vaccines has been shown to increase numbers of Tregs [58, 59]. In the TC-1 model, we observed that rSFVeE6,7 immunization neither led to Treg expansion nor did it affect Treg activity. Additionally, Treg depletion did not enhance the efficacy of the vaccine, implying that in this model, the presence of Tregs has no influence on rSFV-induced immune responses [60].

Radiation therapy using ionizing radiation is a standard treatment for many types of cancer, used most frequently in combination with surgery or chemotherapy. Local tumor irradiation results in the release of tumor antigens and upregulation of MHC I/II, cytokines and chemokines involved in recruitment of T cells to the tumor [61–63]. With an aim to increase T cell homing into the tumor, we combined local tumor irradiation with the rSFVeE6,7 vaccine. In the mouse model, local low-dose tumor irradiation indeed increased homing of antigen-specific CD8^+^ T cells into tumors. When tumor-bearing mice were immunized 1 day following irradiation, this effect was further amplified as compared to irradiation or immunization alone. Concurrently however, irradiation also increased the number of intratumoral MDSCs. Since immunization with rSFV did not further increase the number of MDSCs, the ratio of antigen-specific CD8^+^ T cells to MDSCs in tumors increased 85-fold as compared to the control. The mRNA expression levels of chemokines and their corresponding ligands, such as CCR2 and CCL2, CXCR6 and CCL16, were up-regulated upon irradiation which most likely attributed to homing of different immune cell populations to the tumor. On the whole, this treatment regimen led to a strong increase in the ratio of immune effector cells to immune-suppressive cells, thus skewing towards a more anti-tumor microenvironment [64].

Accumulation of MDSCs in lymphoid organs and tumors is associated with several pathological conditions such as cancer, inflammation and chronic infection [65]. MDSCs are potent suppressors of T-cell responses and to overcome their immune-suppressive activity, we utilized a receptor tyrosine kinase inhibitor, sunitinib malate, due to its capacity to selectively deplete MDSCs and block tumor angiogenesis [66]. We first tested the effect of sunitinib on the levels of MDSCs and observed a dose-dependent decrease in tumor, spleen and circulating blood [67]. Concurrently, sunitinib dose-dependently increased both intratumoral as well as intrasplenic CD8^+^ T cells accompanied with enhanced activation. This was further enhanced on combined treatment of sunitinib with rSFV immunization compared to either treatment alone. The ratio of E7-specific CD8^+^ T cells to MDSCs increased 12.5-fold in comparison with immunization alone and enhanced tumor regression [67]. These results suggest that this combination therapy could be further investigated as a promising approach to improve clinical outcome.

The next step was to combine the above-mentioned strategies, uniting the benefits of single low-dose local tumor irradiation with the therapeutic recombinant SFV-based cancer vaccine and sunitinib [68]. The triple treatment induced the most potent MDSC depletion compared to sunitinib alone. Synchronously, the triple treatment caused an increase in activated and degranulating intratumoral CD8^+^ T cells and further boosted E7-specific CD8^+^ T cells. The triple treatment strongly enhanced the ratio of E7-specific CD8^+^ T cells to MDSCs in tumors with a striking 10,000-fold increase compared to the control tumors. Altogether, the trimodal treatment was successful in blocking tumor development, subsequently leading to 100% tumor-free survival of tumor-bearing mice [68]. The results from this study further strengthen the rationale of employing different treatment modalities to refine cancer immunotherapeutic approaches and bring them closer to the clinic.

With the advent of immune checkpoint blockade, the use of monoclonal antibodies targeting several checkpoint molecules such as PD-1 and CTLA-4, has garnered much interest in cancer immunotherapy. Rice et al. demonstrated that immunotherapy with an adenoviral vector vaccine, encoding the HPV16 E6 and E7 genes, in combination with PD-1 checkpoint inhibition resulted in delayed tumor growth and a significant improvement in survival compared to the controls [69]. Another study by Mkrtichyan and colleagues showed that PD-1 blockade synergizes with depletion of Tregs by low-dose cyclophosphamide in enhancing the therapeutic efficacy of the HPV16 peptide vaccine [70]. A more recent strategy is the dual approach of targeting both inhibitory as well stimulatory molecules. The triple combination of targeting GITR, PD-1 blockade and peptide vaccine induced complete regression in half of the mice, substantially enhancing the anti-tumor immunity [71]. Taking cue from such studies, it would be interesting to investigate the anti-tumor potency of this kind of combination therapy with our vaccine.

## Product and process development

Promising results from our preclinical studies motivated us to test this vaccine in a first-in-man phase I clinical trial. The clinical grade Vvax001 was produced at the Unit Biotech and ATMPs of the Department of Clinical Pharmacy and Pharmacology, University Medical Center Groningen. For application in the trial, the major concern was the scalable production of a clinical batch of rSFVeE6,7, which we named Vvax001. The production comprised electroporation with three different RNAs, using the two-helper system [9] and harvest of the produced viral particles from cultures of electroporated cells. This production process required optimization with respect to safety, scalability and translation to good manufacturing practice (GMP). Vero cells, approved for GMP production, were chosen for the production of the clinical batch (two-helper system). A number of conditions were tested for scalability including different electroporation conditions, optimal temperature and time for harvesting virus after transfection and percentage of fetal bovine serum (FBS). Using the optimized protocol, viral titers of the order 10^8^–10^9^ infectious particles (IP)/ml could be achieved.

A toxicity study was performed in female C57/Bl6 mice using a toxicology batch of Vvax001 produced in a process that was identical to the production process of the clinical grade batch of Vvax001. In addition, tissue distribution and persistence was assessed following a single intra-muscular administration of the highest dose to be tested in subjects, i.e., 5 × 10^8^ IP. No adverse effects were observed in any of the treated animals for the parameters examined. All the immunized mice had HPV16 E7-specific CD8^+^ T cells. Biodistribution/persistence study revealed that the majority of the Vvax001 RNA expressing E6 and E7 of HPV16 was localized at the injection site and was detectable until day 10, which was thereafter rapidly cleared from all tissues. Immunogenicity of the clinical batch with respect to CTL induction and anti-tumor responses was comparable to that of the rSFVeE6,7 vaccine used previously for all preclinical studies. This ‘preclinical’ vaccine was produced on baby hamster kidney cells (BHK-21) using the one-helper system. Altogether, the toxicity and immunization studies attested that the clinical grade Vvax001 was ready for use in the trial.

## Clinical trial

Several clinical trials with VEE-based vaccines have been conducted [72–74], whereas SFV- and SIN-based vector vaccines are yet to be tested in clinic. For example, encouraging results were obtained from a phase I/II clinical trial, conducted to evaluate the safety and CEA-specific immune responses to the immunizations with a VEE replicon particle vaccine, CEA(6D)-VRP (AVX701), in patients with advanced cancer. Repeated administration of the vaccine induced clinically relevant CEA-specific T cell and antibody responses, despite the generation of neutralizing antibodies against the VRP and high levels of Tregs in PBMCs. In addition, patients with CEA-specific T-cell responses appeared to have better survival. Clinical response observed in some patients was attributed to higher dose of VRP-CEA [75].

With respect to HPV, several types of immunotherapeutic approaches aimed at inducing robust cell-mediated responses against HPV-driven infections and malignancies have been developed and tested for safety and efficacy in clinical trials. A phase II randomized, placebo-controlled clinical trial of VGX-3100, a synthetic DNA vaccine targeting HPV 16 and 18 E6 and E7 proteins, in women with CIN2/3 resulted in significantly higher histopathological regression and viral clearance following VGX-3100 vaccination as compared to placebo [76]. This vaccine is currently being investigated in a phase III trial in women with high-grade squamous intraepithelial lesion (HSIL). Therapeutic vaccination with HPV-16 synthetic long peptide vaccine in women with vulvar intraepithelial neoplasia led to durable complete response in 47% of patients [77]. While this vaccine was tested in patients with advanced or recurrent HPV-16 positive gynecological carcinoma, no clinical benefit was observed despite generation of vaccine-induced HPV-16-specific T-cell responses [78]. Furthermore, adoptive T cell transfer of HPV-targeted tumor-infiltrating T cells in women with metastatic cervical cancer resulted in responses in three of nine patients with one partial and two complete responses [79].

Several types of therapeutics examined in trials have rendered varying levels of clinical benefit, which further declines in advanced carcinomas. Owing to several advantages of alphaviral vector vaccine platforms and in view of remarkable preclinical results, we initiated the first-in-human trial of Vvax001 in January 2017. The principal aim of the trial is to evaluate the safety and side-effects as well as immunological activity induced by Vvax001, a therapeutic viral vector vaccine encoding a fusion protein of HPV16-derived E6 and E7 oncogenes. In this phase I study, four dose levels were tested, with three subjects per dose level. The study participants received three doses of the vaccine via intra-muscular injection in an interval of 3 weeks. The side-effects of the vaccine were scored using common toxicity criteria grades. In order to monitor the immune responses, blood was collected at baseline as well as 7–10 days after second and third vaccinations. The immune responses induced by the vaccine were assessed in circulating blood following isolation of peripheral blood mononuclear cells (PBMCs). HPV-16-specific responses to vaccination are being evaluated by a set of immune monitoring assays including interferon-γ ELISPOT (Enzyme-linked ImmunoSpot), intra-cellular cytokine staining (ICS), phenotypic staining as well as proliferation assay for cell-mediated responses and ELISA (enzyme-linked immunosorbent assay) using serum to test for antibodies against the viral vector. The results of this first-in-human trial with a recombinant SFV replicon vaccine will be presented in the second half of 2018.

## Conclusions and perspectives

Alphavirus-based vector systems have several advantages: (1) high heterologous gene expression; (2) induction of apoptosis resulting in cross-presentation of antigens; (3) induction of innate and adaptive immune responses and (4) lack of pre-existing immunity. The combination of all of these features in this vector system results in potent immune responses. We have developed therapeutic vaccines based on SFV vectors. These vaccines have been specially designed, taking into consideration their biosafety for use in humans. Immunization with rSFV induces potent anti-tumor immunity and long-term immune memory in mouse tumor models. The first-in-man trial of Vvax001 will give us further information on its safety, tolerability and immunogenicity in human subjects. On successful completion of the Phase I trial, we would like to evaluate the clinical efficacy of the vaccine with respect to regression of lesions in a Phase II trial. Based on knowledge of its tolerability and therapeutic efficacy in such clinical studies, this vaccine can be extended as a platform to other cancer types.

Combining different therapeutic strategies has emerged as a promising approach towards enhancing anti-tumor efficacy of cancer immunotherapy. Both radiation therapy and sunitinib and their combination can enhance the efficacy of cancer vaccines by modifying the tumor microenvironment supporting tumor eradication. The next approach would be to explore targeted therapies to combine with therapeutic vaccination. This approach will focus on the use of monoclonal antibodies blocking inhibitory checkpoint molecules and/or stimulating co-stimulatory molecules. Comprehensive studies would have to be conducted to attain combination strategies that have a synergistic effect in preclinical models that could be extended to further strengthen anti-tumor efficacy in clinical studies.

In conclusion, we demonstrated that recombinant SFV vectors serve as a suitable vector system for antigen delivery and the vector vaccines that we have developed provide a safe therapeutic tool to combat HCV- and HPV-related infections/malignancies.

## Abbreviations

Ad: Adenovirus
APC: Antigen-presenting cell
CIN: Cervical intraepithelial neoplasia
CTL: Cytotoxic T lymphocyte
CTLA-4: Cytotoxic T lymphocyte antigen-4
DREP: DNA replicon
ER: Endoplasmic reticulum
GITR: Glucocorticoid-induced TNFR-related protein
GMP: Good manufacturing practice
HCV: Hepatitis C virus
HPV: Human papillomavirus
HSIL: High-grade squamous intraepithelial lesion
IP: Infectious particles
MDSC: Myeloid-derived suppressor cell
nsPs: Non-structural proteins
PBMC: Peripheral blood mononuclear cell
PD-1: Programmed cell death protein-1
PRR: Pattern recognition receptor
rSFV: Recombinant SFV
SFV: Semliki Forest virus
SIN: Sindbis virus
Treg: Regulatory T cell
VEE: Venezuelan equine encephalitis virus
VRP: Virus-like replicon particles
